# First person – Rafaela Konstantinidi

**DOI:** 10.1242/bio.062719

**Published:** 2026-06-12

## Abstract

First Person is a series of interviews with the first authors of a selection of papers published in Biology Open, helping researchers promote themselves alongside their papers. Rafaela Konstantinidi is first author on ‘ Investigating the impact of poly(beta amino) ester-mediated *FOXJ1* mRNA delivery on differentiation of primary human bronchial epithelial cells’, published in BiO. Rafaela conducted the research described in this article while a PhD student in Asha Patel, Laura Yates, Clare Lloyd and Sejal Saglani's lab at Imperial College London, London, UK. She is now a scientist at Medicover Genetics, Egkomi, Cyprus, investigating advanced genomic technologies for oncology.

**Figure:**
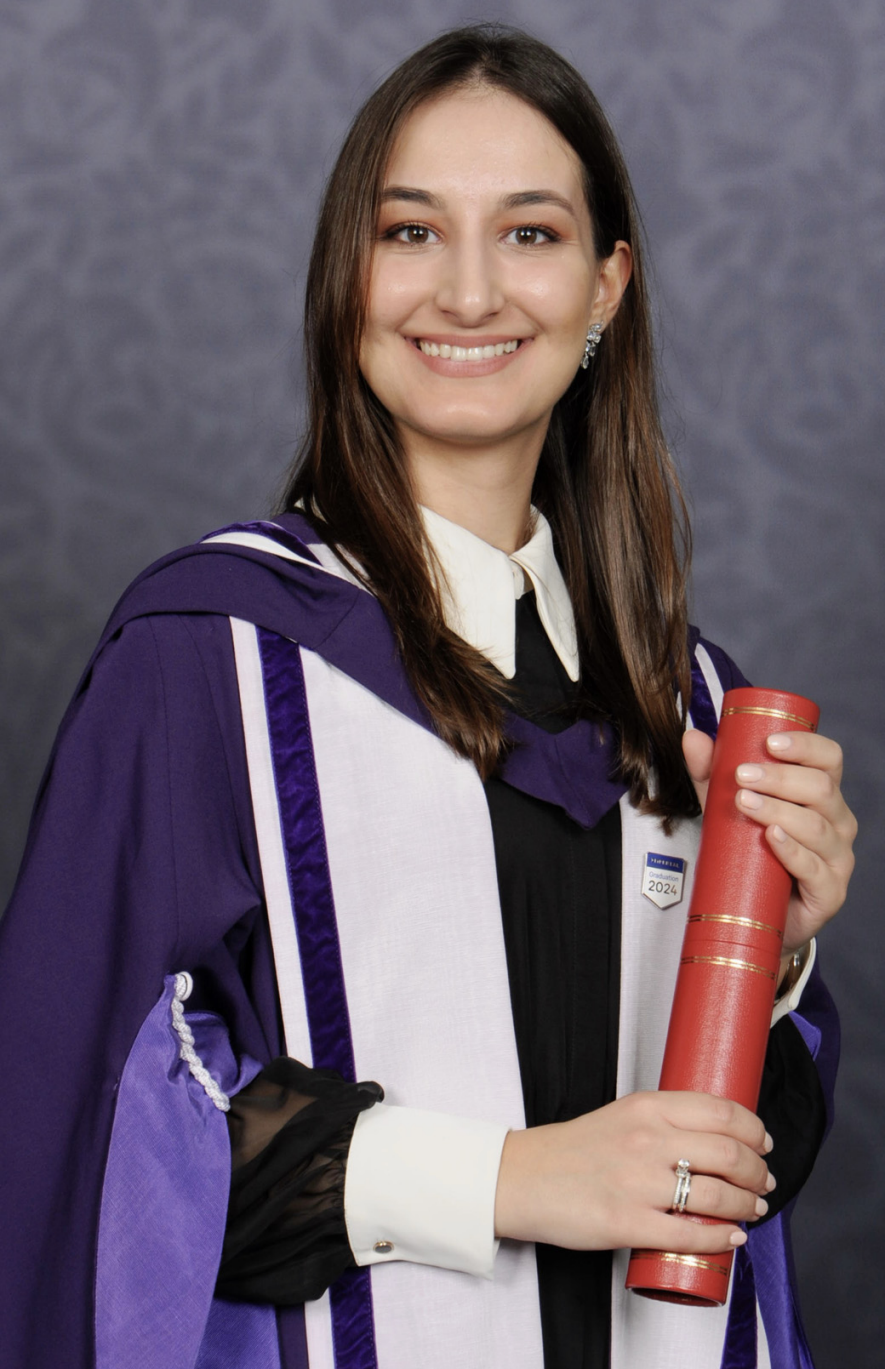
Rafaela Konstantinidi

**Describe your scientific journey and your current research focus**

My scientific journey has been shaped by a strong interest in how molecular and cellular mechanisms can be translated into meaningful advances in human health. I began by building a broad foundation in biomedical science and novel therapies, before focusing my PhD at Imperial College London on nanoparticle-mediated mRNA delivery and its effects on primary human bronchial epithelial cell differentiation. This work strengthened my expertise in complex *in vitro* models, nucleic acid delivery, molecular biology and translational research. I then expanded my experience in industry research and development (R&D) at AstraZeneca UK, where I contributed to respiratory and immunology programmes across the R&D pipeline, from early-stage discovery prior to candidate drug nomination to projects already progressing through clinical development. I am currently part of the Oncology Core team within the R&D department at Medicover Genetics in Cyprus, where my research focuses on developing and validating screening and diagnostic methods for cancer. This includes assay development, technical and clinical validation, and the evaluation and interpretation of results to support high-quality genetic diagnosis and clinical decision-making for cancer patients.

**How would you explain the main finding of your paper?**

The main finding of our paper is that we can give airway cells temporary genetic instructions, using a molecule called mRNA, without affecting their ability to mature into a healthy airway lining. These cells are essential for maintaining and repairing the airways, where they help form a protective surface that traps and clears particles, mucus and other unwanted material from the lungs. We investigated whether delivering this transient genetic message into the cells would interfere with their normal development. Reassuringly, it did not. The cells received the message, produced the intended protein and then continued to mature as expected. Once fully developed, the treated cells formed an organised airway lining that closely resembled untreated cells, including the specialised cell types needed for normal airway function. Overall, our study shows that mRNA can be used to deliver transient genetic instructions to airway cells without compromising their ability to develop properly. This provides a transient tool for studying how airway cells grow, specialise and maintain a functional airway surface.

Overall, our study shows that mRNA can be used to deliver transient genetic instructions to airway cells without compromising their ability to develop properly

**Figure:**
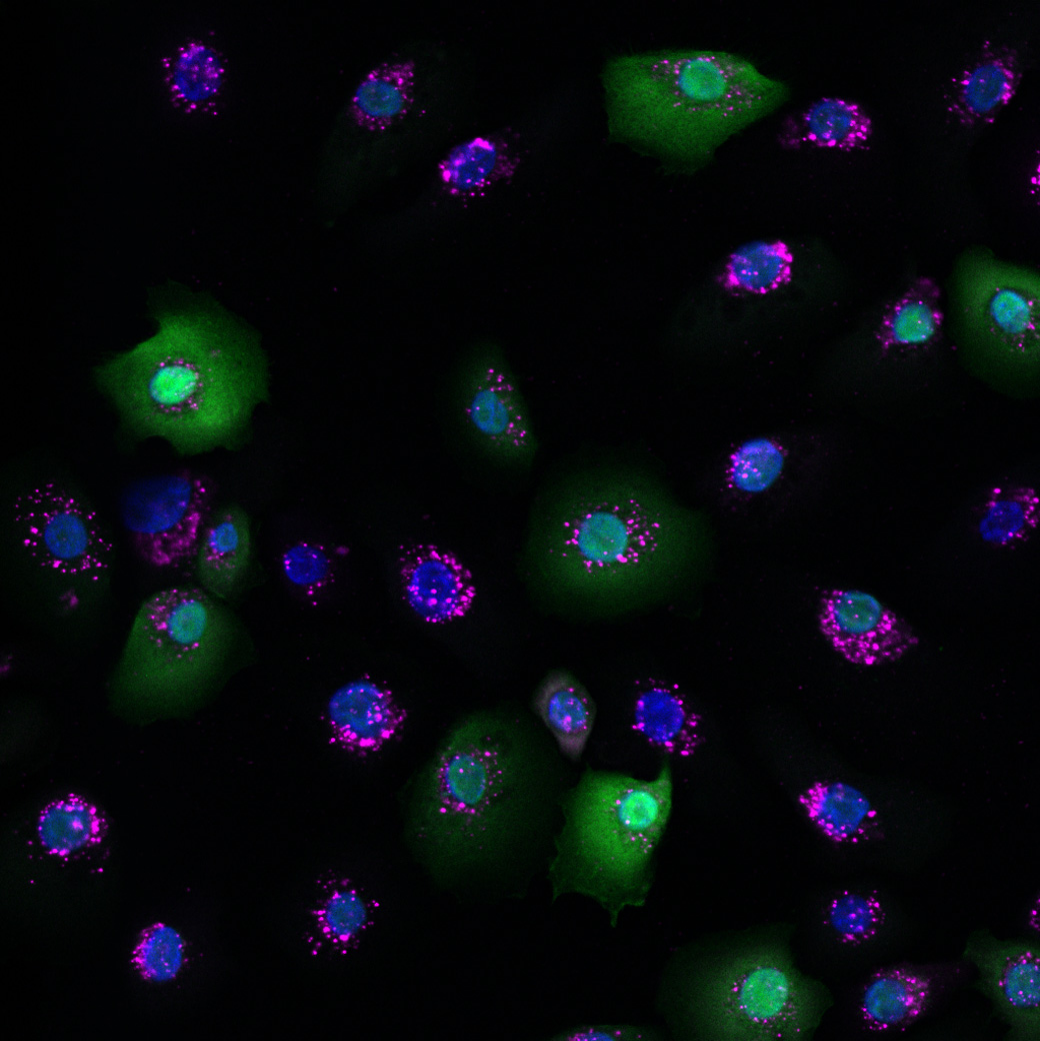
**Message received: primary human bronchial epithelial cells translate mRNA into protein.** Fluorescence microscopy image of primary human bronchial epithelial cells from a healthy donor, a cell type that is challenging to transfect. Cell nuclei are shown in blue, mRNA uptake in magenta, and successful translation of GFP mRNA into GFP protein in green.

**What are the potential implications of this finding for your field of research?**

Our findings suggest that mRNA delivery can be used as a novel, transient approach to study gene function in primary human bronchial epithelial cells without compromising their ability to form a mature, mucociliary epithelium. This is important for the respiratory field because it shows that researchers can modulate the expression of key genes in basal progenitor cells while still following their subsequent differentiation in a physiologically relevant air-liquid interface model. Our work also provides evidence that poly(beta amino) ester nanoparticles, as a non-viral delivery system, can deliver mRNA effectively to primary human bronchial epithelial cells while preserving epithelial integrity and differentiation. More broadly, these findings could help expand the use of mRNA technologies in respiratory biology, both as tools to investigate airway repair, regeneration and disease mechanisms, and as platforms for testing how transient gene manipulation affects epithelial cell behaviour. In the longer term, this work may contribute to the development of more targeted RNA-based strategies for studying and potentially targeting airway diseases.

… meaningful progress often comes from persistence, patience and learning from setbacks

**What piece of advice would you give to the next generation of researchers?**

My advice to the next generation of researchers would be to stay curious, resilient and open-minded. Research rarely follows a straight path, and some of the most valuable lessons come from experiments that do not work or results that challenge our expectations. I would encourage young researchers to build a strong technical foundation, but also to develop skills in communication, collaboration and critical thinking, as science increasingly depends on working across disciplines. Most importantly, choose questions that excite you, remain adaptable as your interests and opportunities evolve, and remember that meaningful progress often comes from persistence, patience and learning from setbacks.
